# Metabolic dysfunction‐associated steatotic liver disease is associated with accelerated brain aging: A large population‐based study

**DOI:** 10.1002/alz70860_102203

**Published:** 2025-12-23

**Authors:** Rongrong Yang

**Affiliations:** ^1^ Karolinska Institutet, Stockholm, Sweden, Sweden; Tianjin University of Traditional Chinese Medicine, Tianjin, Tianjin, China

## Abstract

**Background:**

Metabolic dysfunction‐associated steatotic liver disease (MASLD) has been linked to cognitive decline and dementia risk. We aimed to investigate the association between MASLD and brain aging and explore whether low‐grade inflammation plays a role in this association.

**Method:**

Within the UK Biobank cohort, 30,386 chronic neurological disorders‐free adults, who underwent brain magnetic resonance imaging (MRI) scans an average of 9 years after baseline, were included. Individuals were categorized into no MASLD/related SLD and MASLD/related SLD (including subtypes of MASLD, MASLD with increased alcohol intake [MetALD], and MASLD with other combined etiology). Brain age was estimated using machine learning algorithms based on 1,079 brain MRI phenotypes. Brain age gap (BAG) was calculated as the difference between brain age and chronological age. Low‐grade inflammation was calculated by inflammation score (INFLA) based on white blood cell count, platelet, neutrophil granulocyte to lymphocyte ratio, and C‐creative protein. Data were analyzed using linear regression and structural equation models.

**Result:**

At baseline, 7,360 (24.2%) participants had MASLD/related SLD. Compared to participants with no MASLD/related SLD, those with MASLD/related SLD had significantly larger BAG (β = 0.86, 95% CI = 0.70, 1.02), as well as those with MASLD (β = 0.59, 95% CI = 0.41, 0.77) or MetALD (β = 1.57, 95% CI = 1.31, 1.83). The association between MASLD/related SLD and larger BAG was significant across middle‐aged (<60) and older (≥60) adults, males and females, and *APOE* ɛ4 carriers and non‐carriers. INFLA mediated 13.53% of the association between MASLD/related SLD and larger BAG (*p* < 0.001).

**Conclusion:**

MASLD/related SLD is associated with accelerated brain aging, even among middle‐aged adults and *APOE* ɛ4 non‐carriers. Low‐grade systemic inflammation may partially mediate this association.

